# Psychopathological symptoms among the russian population during the COVID-19 pandemic in the spring of 2020

**DOI:** 10.1192/j.eurpsy.2021.821

**Published:** 2021-08-13

**Authors:** J. Koniukhovskaia, E. Pervichko, O. Stepanova, E. Dorokhov

**Affiliations:** 1 Psychology, Lomonosov Moscow State University, Moscow, Russian Federation; 2 Faculty Of Psychology, Lomonosov Moscow State University, Moscow, Russian Federation; 3 Faculty Of Psychology And Social Sciences, Pirogov Russian National Research Medical University, Moscow, Russian Federation

**Keywords:** COVID-19, Psychopathological symptoms, SCL -32

## Abstract

**Introduction:**

The COVID-19 pandemic has affected the lifestyle and psychological well-being of millions of people.

**Objectives:**

The aim of the study was to assess the prevalence of psychopathological symptoms in the Russian population in the COVID- 19 pandemic context.

**Methods:**

We used a socio-demographic questionnaire (20 questions) and a Short Scale for Psychopathological Symptom Checklist (SCL -32) (Derogatis 1977; Mitina, Gorbunova, 2011). 582 Russian residents (496 women and 86 men) aged 18-64 years participated in the online survey in May 2020.

**Results:**

Women were significantly more likely than men to have somatic dysfunctions (5.6±2.54vs4.8±1.9;p=0.001), interpersonal problems (6.97±2.9vs6.0±2.8; p=0.005), depression signs (6.9±3.2vs5.7±2.9;p=0.001) and anxiety disorders (6.8±2.8vs5.2 ±2.1;p=0.000), as well as sleep disorders (6.4±2.8vs5.9±2.1;p=0.049) and suicidal thoughts (4.2±1.8vs3.7±1.8;p=0.032). In addition, women are more hostile than men are (6.3±2.7 vs 5.1±2.3; p=0.000). Respondents under the age of 30 are more likely than older people to have interpersonal problems (p=0.286, p=0.000), as well as signs of depression (p=0.216, p=0.000), hostility (p=0.226, p=0.000) and psychoticism (p=0.203, p=0.000). Respondents’ low income is statistically associated with interpersonal problems (p=0.139,p=0.001), anxiety (p=0.131, p=0.002), hostility (p=0.156, p=0.000), psychoticism (p=0.137, p=0.001), and suicidal intentions (p=0.152,p=0.000). Among respondents whose relatives had COVID-19, signs of anxiety disorders (7.2±3vs3.5±2.9;p=0.027) and obsessive disorders (8.1±3.2 vs 7.3±2.6; p=0.029) were significantly more common than in the whole sample.

**Conclusions:**

The study highlights socio-demographic factors of vulnerability to psychopathological symptoms in the COVID-19 pandemic context, which should be taken into account when organizing medical and psychological assistance to the population.

